# 

**Published:** 1972-07

**Authors:** 


Bristol Medico-Chirurgical Journal Vol. 87 (iii) No. 323

### Contents

Fifty Years of Brentry Hospital .................... 23

J. Jancar

The General Practitioner Barium Meal Service ... 31
C. Johnson and J. B. Penry

The Red Cell Volume in a Hospital Population ... 33
C. D. R. Pengelly and Vivien McKenzie

Printed by F. Bailey & Son Ltd., Dursley, Glos.

